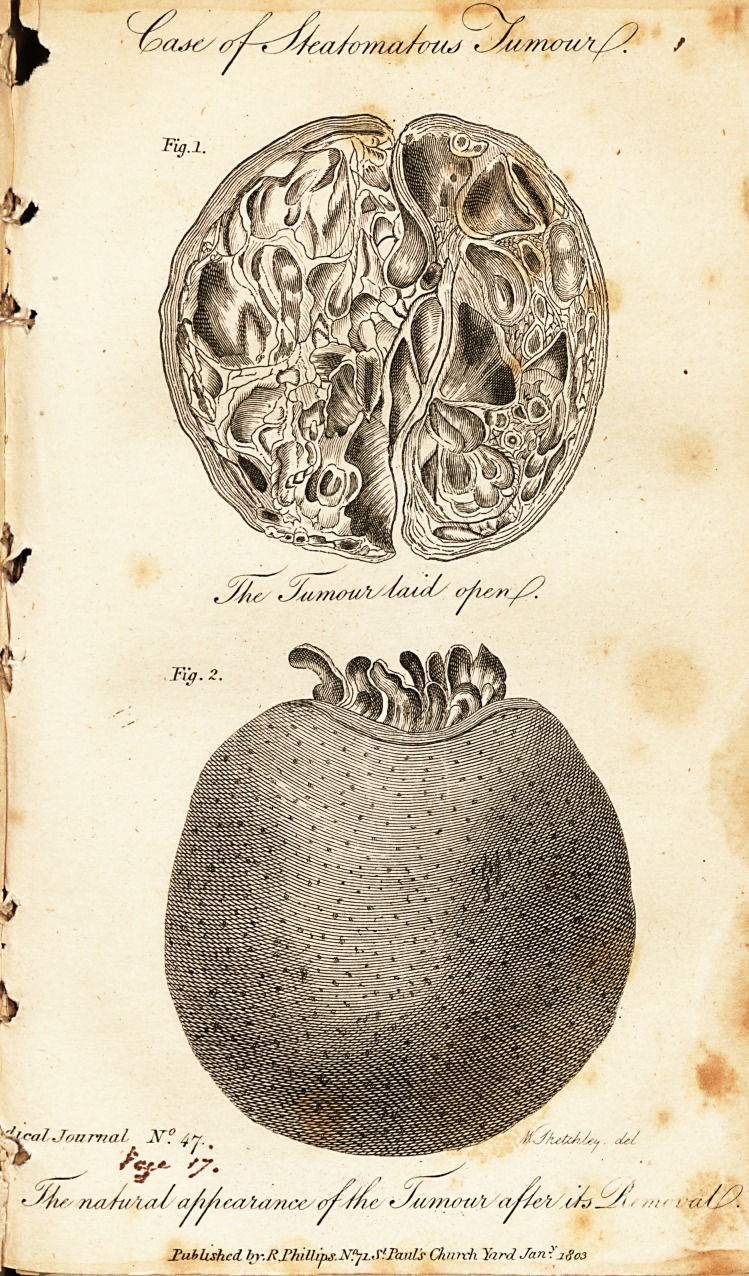# Mr. Hutchinson's Case of Steatomatous Tumour

**Published:** 1803-01-01

**Authors:** Benjamin Hutchinson

**Affiliations:** Southwell, Notts


### *To the Editors of the Medical and Phyſical Journal.*

GENTLEMEN,

PRETERNATURAL enlargements in various parts of the human frame being the frequent subjects of surgical treatment, the following Case of Steatomatous Tumour may not be deemed unimportant. The size and situation of this tumour being unusual, I have obtained, on a diminished scale, a faithful delineation of its appearance immediately on its removal, and when laid open, *(see the plate).* I am greatly indebted to my ingenious friend, Mr. Sketchly, of this place, for this representation of it, and which I imagine you will think deserving a place in your truly valuable Journal.

EDWARD FEATHERSTONE, a cottager residing at Maplebeck, a village in this neighbourhood, a man of robust and healthy stamina, and between fifty and sixty years of age, about seventeen years ago, perceived a small swelling somewhat resembling a wart, situated on the left side of the raphe of the perinæum. The tumour rapidly increasing for some years, he applied to an empiric, who made several unsuccessful attempts to reduce its size by various applications calculated to promote absorption. In the course of the last summer, my patient suffering considerable pain from an excoriation of this excrescence, and still greater inconvenience from its suspension and weight, (the size of it being now equal to that of a child's head of seven years old) he requested my assistance in healing the small superficial ulcerations. Imagining the tumour to be of the steatomatous species, and that it received its supply of blood from the vascular organization of the bulbous portion of the urethra, I encouraged the poor man to submit to its removal; a proposal to which he very cheerfully consented; and on the eleventh of last month, in the presence of Mr. BECHA, formerly a respectable Surgeon in this place, and Mr. PIGOT my pupil, the operation was performed, previously securing my patient on a table as in performing lithotomy. To prevent embarrassment from profuse hæmorrhage, I applied the stick tourniquet around the neck of the tumour; by which compression of the vessels, the operation was not attended with the least difficulty. An invariably attendant circumstance with enlargements of this nature is, an abundant vascular supply; and I suppose that not fewer than twenty arteries were secured by ligatures: the lips of the wound were approximated as near as possible, the common dressings were secured by the T bandage, and my patient recovered without any interruption. Four pounds and a half was the weight of this tumour on its removal, and its diameter about seven inches.

Preternatural tumours are supposed to be either rapid or slow in their progressive steps of increase, nearly in proportion to the degree of inflammation with which they are attended. We may therefore conclude that the hasty progress and increase in the case above related, may be attributed to its peculiar situation, exposing the excrescence to the constant irritation of friction and consequent inflammation; and there exists but little doubt in my mind, that had it remained some years longer, it might have attained the weight of at least one sixth of the whole body, reaching probably from the perinæum to the ground, instances of which are recorded in the Annals of Surgery.

Great attention is requisite in distinguishing the nature and contents of tumours previous to their being opened or removed. Systematic writers on Surgery describe steatomatous tumours as being invariably of a firm consistence, as rolling more readily under the skin than others, and their surface as being unequal. I would wish to observe, that these marks of distinction are by no means to be considered as constantly existing. In the case above described, the feel of the tumour conveyed the idea of some fluid fluctuating under the fingers, and its surface was perfectly smooth; circumstances which might have led me into an erroneous opinion of the nature of its contents, had I not met with two or three similar instances, one of which I successfully recovered.

I am, &c.

*Southwell, Notts.*
*Dec. 3, 1802.*

BENJAMIN HUTCHINSON.

---

DESCRIPTION of the PLATE.

Fig. 1. The tumor laid open.

Fig. 2. The natural appearance of the tumour after its removal.

*Observations*

### Case of Steatomatous Tumour.

Fig. 1.

The Tumour laid open.

Fig. 2.

## Figures and Tables

**Fig.1. Fig. 2. f1:**